# Reconstitution of immune cell interactions in free-standing membranes

**DOI:** 10.1242/jcs.219709

**Published:** 2018-10-02

**Authors:** Edward Jenkins, Ana Mafalda Santos, Caitlin O'Brien-Ball, James H. Felce, Martin J. Wilcock, Deborah Hatherley, Michael L. Dustin, Simon J. Davis, Christian Eggeling, Erdinc Sezgin

**Affiliations:** 1 MRC Human Immunology Unit, Weatherall Institute of Molecular Medicine, University of Oxford, Oxford, OX3 9DS, UK; 2 Kennedy Institute of Rheumatology, University of Oxford, Oxford, OX3 7FY, UK; 3 Institute of Applied Optics Friedrich-Schiller-University Jena, Max-Wien Platz 4, 07743 Jena, Germany; 4 Leibniz Institute of Photonic Technology e.V., Albert-Einstein-Straße 9, 07745 Jena, Germany

**Keywords:** Immune signalling, Immune synapse, *In vitro* reconstitution, Model membranes, Giant unilamellar vesicles

## Abstract

The spatiotemporal regulation of signalling proteins at the contacts formed between immune cells and their targets determines how and when immune responses begin and end. Therapeutic control of immune responses therefore relies on thorough elucidation of the molecular processes occurring at these interfaces. However, the detailed investigation of each component's contribution to the formation and regulation of the contact is hampered by the complexities of cell composition and architecture. Moreover, the transient nature of these interactions creates additional challenges, especially in the use of advanced imaging technology. One approach that circumvents these problems is to establish *in vitro* systems that faithfully mimic immune cell interactions, but allow complexity to be ‘dialled-in’ as needed. Here, we present an *in vitro* system that makes use of synthetic vesicles that mimic important aspects of immune cell surfaces. Using this system, we began to explore the spatial distribution of signalling molecules (receptors, kinases and phosphatases) and how this changes during the initiation of signalling. The GUV/cell system presented here is expected to be widely applicable.

## Abstract (precis)

**Summary:** Immune cell–cell interactions are reconstituted in free-standing vesicles, allowing the spatiotemporal aspects of immune synapse formation to be investigated.

## INTRODUCTION

Dynamic cell–cell contacts govern the activation and effector functions of immune cells. Communication occurs through membrane protein interactions on opposing surfaces, whereby surface-presented antigens and ligands are recognised by key immune cell receptors. This induces intracellular signalling cascades that lead, eventually, to the formation of an immunological synapse, which comprises a spatiotemporally regulated supramolecular cluster of proteins at the interface between the cells (Dustin and Baldari, 2017; Dustin and Choudhuri, 2016). Quantitative investigation of the receptors and their molecular behaviour at the cellular contact is essential in order to understand how immune cells integrate activating and inhibitory signals, allowing decisions about whether/when to respond (Dustin and Groves, 2012; Kamphorst et al., 2017). Studying these factors in physiological systems is, however, challenging because of the topographical complexity and transient nature of immune cell–cell contacts. In addition, surface protein dynamics and organisation can be influenced by a variety of factors such as protein–protein or protein–lipid interactions, the activity of the cortical actin cytoskeleton and the barrier properties of the glycocalyx, which makes it challenging to identify the exact role of each component (Chernomordik and Kozlov, 2003; Cho and Stahelin, 2005; Lemmon, 2008; Ritter et al., 2013). To this end, minimal *in vitro* systems with controllable complexity are essential tools for unravelling the molecular biology of cell–cell contact.

The most basic systems for reconstituting immune cell interactions are planar substrates coated with immobile antibodies or purified biological ligands (Bunnell et al., 2001). Glass-supported lipid bilayers (SLBs) reconstituted with mobile proteins acting as surrogate antigen-presenting cell (APC) surfaces capture additional features of physiological T cell–APC interfaces (Dustin et al., 2007). Advantages of SLBs include being able to control protein variety and density, and a two-dimensional format that allows advanced optical imaging of the contact. Accordingly, SLBs have been used extensively to study immune cell activation (Bertolet and Liu, 2016; Dustin et al., 2007; Lever et al., 2016; Lopes et al., 2017; Zheng et al., 2015). However, use of solid supports and SLBs also has several disadvantages. First, the small hydration layer (1–2 nm) between the bilayer and the underlying support is insufficient to completely de-couple the support's influence on reconstituted proteins: the glass support restricts diffusion of the molecules in the membrane plane, mostly in an unpredictable manner, thereby affecting the membrane dynamics significantly (Przybylo et al., 2006; Sezgin and Schwille, 2012) and influencing cell behaviour (Sánchez et al., 2015). Second, the solid glass support imposes rigidity on the lipid membrane. Although it varies, the stiffness of immune cell membranes is known to be several orders of magnitude lower than that of SLBs, that is, 0.1–1 kPa versus 1 MPa for SLBs (Bufi et al., 2015; Rosenbluth et al., 2006; Saitakis et al., 2017), and it has been shown that substrate stiffness influences B- and T-cell migration, synapse formation and signalling (Judokusumo et al., 2012; Martinelli et al., 2014; Natkanski et al., 2013; Schaefer and Hordijk, 2015; Shaheen et al., 2017; Tabdanov et al., 2015; Zeng et al., 2015). Third, the necessarily large area and planar nature of SLBs (i.e. centimetres) mean that they are poor mimics of the topological constraints experienced by cells *in vivo*, although this can be somewhat overcome by nanofabrication methods that partition bilayers (Choudhuri et al., 2014).

A simple alternative to an SLB is the giant unilamellar vesicle (GUV). Vesicular systems are not influenced by any surface (i.e. they are free standing) and suitably mimic cells with respect to their finite size (10–100 µm diameter), flexibility, deformability, stiffness and free membrane fluctuations (Fenz and Sengupta, 2012; Schmid et al., 2015). However, similarly to SLBs, they can be engineered to have various lipid compositions and to present membrane proteins, further emulating physiological membranes. GUVs are also amenable to light microscopy-based imaging. Very recently, GUVs were used for the first time to mimic T cells interacting with SLBs as the surrogate APC surface (Carbone et al., 2017). Minimal systems of this type (GUV–SLB) are likely to be especially informative with respect to understanding how spontaneous, topologically driven processes shape the spatiotemporal properties of cell–cell contacts. At the next level, the analysis of live cells interacting with GUVs offers a way to dissect both passive and active processes driving the nascent immune response.

In this study, we utilised GUVs reconstituted with cell surface proteins as controllable, reductionist mimetics of APCs. Using this approach, we explored the interactions of live T cells, B cells and mast cells with GUVs, directly observing the types of reorganisation of signalling proteins at the contacts that could explain the earliest stages of their activation, including kinase recruitment. We also exploited the tuneable nature of GUVs to study the topological basis of protein reorganisation at the model contacts, highlighting their versatility. We anticipate that GUVs will become an increasingly popular tool for studying contact-dependent immune cell interactions.

## RESULTS

### Preparation and characterisation of protein-loaded GUVs

**Fig. 1.**
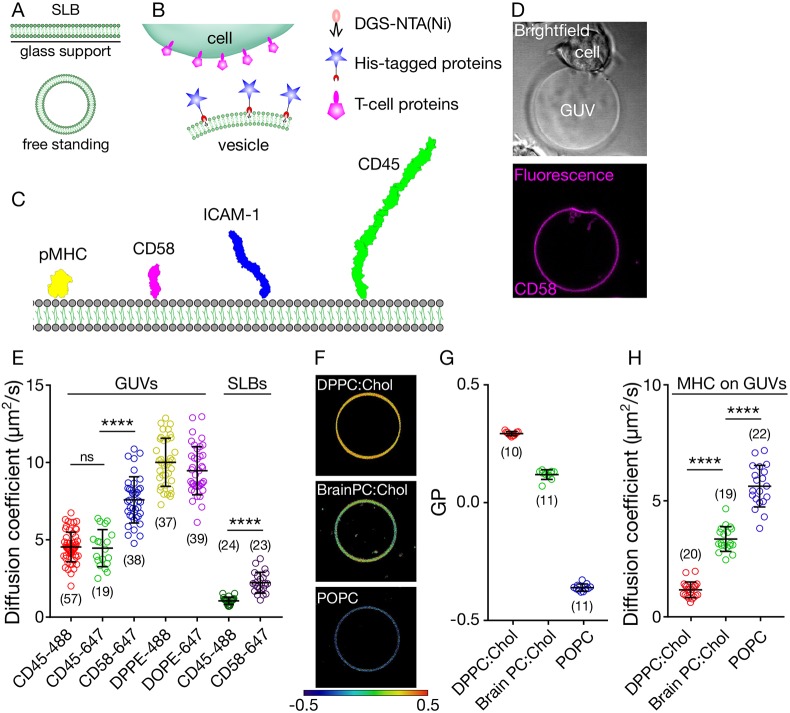
**The *in vitro* system.** (A) Depiction of supported lipid bilayers and free-standing vesicles. (B) Scheme showing the *in vitro* cell–vesicle interaction. (C) Molecules of interest for this study, drawn to scale based on structure determinations (Chang et al., 2016). (D) Example bright field (top) and fluorescence (bottom) images of CD2^+^ Jurkat–CD58^+^ GUV contact (image size 50 µm×50 µm). (E) Diffusion analysis of fluorescently labelled lipids and proteins in GUVs and SLBs. (F) Lipid packing of GUVs of varying composition revealed by a GP map (image size 40 µm×40 µm). (G) Quantification of the GP. (H) Diffusion analysis of fluorescently labelled pMHC on GUVs composed of different lipids. Student's *t*-test (two-tailed) was used to determine significance (****P<0.0001). Error bars represent standard deviation of the mean. Number of data points obtained from at least three independent experiments are indicated on the graphs in parentheses.

SLBs are membrane bilayers sitting on top of a glass support with a thin water layer sandwiched between (Fig. 1A). Reconstitution of proteins on SLBs yields an effectively infinite flat surface that can be used as a mimic of an immune cell surface. Free-standing membranes, on the other hand, are freely floating vesicles that are not influenced by any surface (Fig. 1A) and have a finite size (typically tens of micrometres). Here, we explore the use of free-standing GUVs as a model system to mimic the immune cell surface.

To attach immune cell surface proteins to the GUV surface, we prepared GUVs with a small fraction (4 mol%) of nickel-nitrilotriacetic acid (Ni-NTA)-functionalised lipids capable of directly binding to His-tagged surface proteins, each of which had been expressed in a soluble form. To investigate protein interactions at the model cell–cell contact, we used His-tagged versions of proteins that are known to be involved in immune cell activation (Fig. 1B), such as peptide/major histocompatibility complex (pMHC), cluster of differentiation (CD) 45, intercellular adhesion molecule 1 (ICAM-1) and CD58 (Fig. 1C). With either pMHC, which binds the T-cell receptor (TCR), or CD58, which binds the small adhesion protein CD2 on the surface of T cells, presented on the GUVs, we could create specific cell–vesicle contacts wherein these proteins would accumulate (Fig. 1D). We did not, however, observe protein accumulation for GUVs not presenting cognate His-tagged protein (Fig. S1).

As discussed above, reduced protein diffusion caused by interaction with the glass support is a drawback of the SLB system. Given that interactions at the cell surface are diffusion limited once the membranes are in close proximity (Blouin et al., 2016; Sánchez et al., 2015; Sezgin et al., 2017; Veya et al., 2015), we were interested in characterising the diffusivity of the proteins on GUVs compared with SLBs. We measured the diffusion coefficients of proteins of interest using fluorescence correlation spectroscopy (FCS), positioning the confocal spot on the membrane in SLBs or at the bottom of the GUVs (Fig. S1). First, we tested whether the fluorescent tags had any influence on protein mobility by comparing the diffusion coefficient of CD45 with different fluorophores on GUVs (Alexa Fluor 488 and 647). Both labelled forms of these molecules exhibited similar diffusion (Fig. 1E). We then determined how the diffusion of proteins was affected by their size. Unsurprisingly, the smaller protein CD58 diffused much faster (almost as fast as lipids) than the larger protein CD45 (Fig. 1E; for structure-based size comparisons, see Fig. 1C). We next compared the diffusion of these proteins on GUVs versus SLBs. Strikingly, the diffusion of CD45 and CD58 was significantly slower (about one-fifth) on SLBs than on GUVs, presumably caused by the influence of the glass support (Fig. 1E).

Altered diffusion (i.e. in more rigid membranes) is known to be achievable by varying the lipid composition of the vesicles (Macháň and Hof, 2010). POPC is a phospholipid that bears chains of saturated palmitic acid (16 carbon; 16:0) and mono-unsaturated oleic acid (18 carbon; 18:1). Therefore, membranes composed of POPC are relatively fluid. Fluidity can be measured empirically using an index called generalised polarisation (GP) and polarity sensitive dyes (Sezgin et al., 2015b). A GP map reveals the relative level of lipid packing of membranes, with GP varying between −1 (maximally disordered; dark blue) and +1 (maximally ordered; dark red, see Materials and Methods for details of GP imaging). The fluidity of POPC GUVs, for example, is revealed by its blue colour in the GP map (Fig. 1F,G). Brain PC (Avanti Polar Lipids) is a mixture of phosphatidylcholine (PC) lipids (saturated and unsaturated), which, together with cholesterol (Chol; which orders the membrane when present alongside unsaturated lipids), yields a membrane of intermediate fluidity (Fig. 1F,G; yellow in the GP map). By contrast, dipalmitoylphosphatidylcholine (DPPC) carries two saturated palmitic acids, leading to the formation of a more rigid membrane. DPPC alone forms a gel phase, which is usually not found in biological systems. However, when cholesterol is present, DPPC forms a liquid phase with relatively low fluidity (Fig. 1F,G; red in the GP map).

To test the effects of the three membrane systems (POPC, Brain PC:Chol and DPPC:Chol) on protein diffusivity, we inserted the ligand of the TCR (pMHC) into the three types of GUVs. We found that the pMHC complex diffused as rapidly as CD58 in POPC membrane (consistent with their similar sizes). Its diffusion was slower in BrainPC:Chol and further reduced in DPPC:Chol membranes, where it approached the rate of diffusion of proteins on SLBs (Fig. 1H). These observations show that, if required, more-saturated lipid mixtures allow for slower yet unhindered diffusion in free-standing GUVs, in contrast to the default slow diffusion observed in SLBs that results from interference by the support. Hereafter, we used GUVs made only of POPC.

### Spatiotemporal reorganisation of cell surface proteins at cell–GUV contacts

The spatial organisation of signalling proteins at the cell–cell contact has been of great interest because of its probable role in the initiation of lymphocyte activation. Immune signalling events usually start with the phosphorylation of a receptor such as the TCR, B-cell receptor (BCR) or Fc receptors (FcRs) by intracellular kinases such as Lck or Lyn (Brownlie and Zamoyska, 2013). It has been argued that the size-based exclusion of CD45 from contacts crucially breaks the local balance between phosphatases and kinases, allowing the kinases to initiate signalling (Davis and van der Merwe, 2006). Consistent with this idea, structural work on CD45 indicates that even the smallest form of CD45 is larger than the complex formed by the TCR and its pMHC ligands (Chang et al., 2016). However, there have been other explanations for how the segregation of CD45 might occur *in vivo*, such as partitioning into lipid domains (Stone et al., 2017), charge effects (Su et al., 2016), or interactions of CD45 with active diffusional barriers created by integrins (Freeman et al., 2016).

**Fig. 2.**
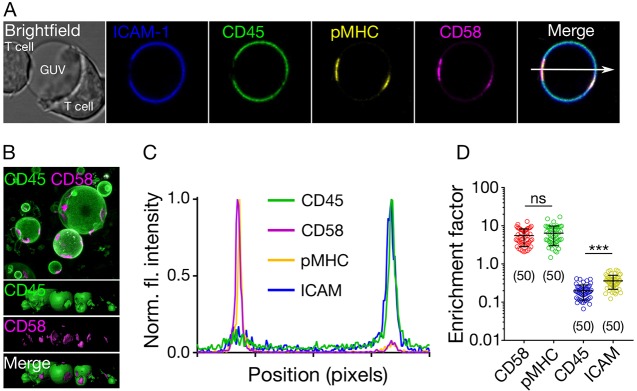
**Protein reorganisation at cell**–**GUV contacts.** (A) Distribution of ICAM-1, CD45, pMHC and CD58 at cell–GUV contacts (image size 40 µm×40 µm). (B) 3D image (top, top view of raw image; bottom*,* side views of surface image) of the contact formed between 1G4 T cells and GUVs, showing the abundance of contacts formed (image size 75 µm×75 µm). (C) Intensity line profile (arrow shown in A) of the fluorescence signal through the T cell contacting the GUV. (D) Quantification of the fluorescence signal inside and outside of the contacts (inside/outside ratio) for all four proteins. Student's *t*-test (two-tailed) was used to determine significance (***P<0.001). Error bars represent standard deviation of the mean. Data are representative of at least three independent experiments and for each data set, the number of data points is indicated on the graphs in parentheses.

We used the *in vitro* GUV-based system to investigate the principles of protein spatial organisation at cell–cell contacts in three dimensions. We used a 1G4 TCR-expressing Jurkat T cell line to study the formation of contacts between cells and vesicles presenting the His-tagged proteins shown in Fig. 1C, using the NTA-His coupling method depicted in Fig. 1B. These proteins were: (1) the pMHC recognised by the 1G4 TCR (i.e. a peptide derived from the tumour antigen NY-ESO; Chen et al., 2005); (2) CD58, which is the ligand of the small adhesion protein CD2; (3) ICAM-1, which binds to LFA-1; and (4) the phosphatase CD45, which is expressed by lymphocytes and APCs. The proteins were fluorescently labelled; colours are depicted in Fig. 1C (see Materials and Methods). We observed enrichment of pMHC and CD58 in the region of contact between the 1G4^+^ T cell and the GUV, as expected (Fig. 2A). The larger molecules ICAM-1 and CD45 were, in contrast, excluded from the contact (Fig. 2A, Fig. S2). The segregation of CD45 and ICAM-1 from pMHC and CD58 was confirmed in three-dimensional images of the contacts in a large field of view (Fig. 2B). Fluorescence intensity line-profiling across the GUV was used to quantify the changes (Fig. 2C; line shown in Fig. 2A). The ratio of the two peaks in the line profile (i.e. fluorescence at the contact side versus the non-contact side) was used to calculate the ratio of protein inside and outside the contact (the enrichment factor). A value of one for the enrichment factor represents no preference, and values >1 and <1 represent enrichment and exclusion from the contact, respectively. The enrichment factor was ∼5–8 for CD58 and pMHC, ∼0.2 for CD45 and ∼0.4 for ICAM-1, confirming that CD45 and ICAM-1 are readily excluded from contacts enriched with CD58 and pMHC (Fig. 2D). The slightly higher, yet statistically significant exclusion of CD45 over ICAM-1 (Fig. 2D) was expected because of their difference in size (Fig. 1C).

**Fig. 3.**
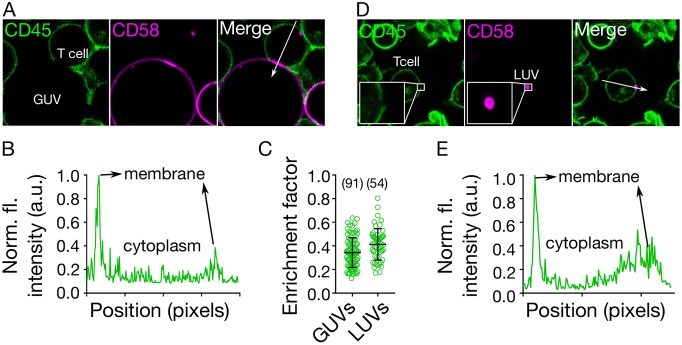
**Requirements for CD45 segregation.** (A) Distribution of CD45 (on the T cell surface) and CD58 (attached to the GUV), showing that close contact induces local exclusion of CD45 phosphatase (image size 40 µm×40 µm). (B) Line plot of CD45 fluorescence intensity indicated by white arrow in A. (C) CD45 exclusion at GUV/LUV–cell contacts. The enrichment factor represents the ratio of fluorescence intensity at the contact site versus non-contact site. (D) Small LUVs (<250 nm, coated with CD58) induce exclusion of CD45 on cells (image size 50 µm×50 µm). (E) Line plot of CD45 fluorescence intensity indicated by white arrow in D. Cells were labelled with anti-CD45 Gap8.3 Fab-Alexa Fluor 488. Error bars represent standard deviation of the mean. Data is representative of at least three independent experiments; for each data set, the number of data points is indicated on the graphs in parentheses.

To determine whether the pMHC–TCR interactions were needed for the exclusion of CD45, and whether T-cell-expressed CD45 was also excluded from the contact with the GUVs, we used GUVs presenting only CD58, and labelled CD45 on the T cells with the fragment antigen-binding (Fab) of Gap8.3 anti-CD45 antibody, labelled with Alexa Fluor 647 fluorescent dye. CD45 was excluded from regions of T cell-GUV contact where CD58 accumulated (Fig. 3A–C, Fig. S3).

### Dependence of spatiotemporal reorganisation on contact size

The initial interaction between T cells and their targets is proposed to be via T cell microvilli (Cai et al., 2017; Jung et al., 2016). However, it was unclear if this would lead to CD45 segregation. The diameters of the tips of microvilli are believed to be in the region of 100–200 nm, which is close to the limit of diffraction-limited imaging. One advantage of the free-standing vesicle system is that vesicles are readily tuneable in size. We prepared CD58-presenting large unilamellar vesicles (LUVs) of size <250 nm to determine whether CD45 would segregate at contacts formed at these lengths scales. We readily observed segregation on T cells interacting with the vesicles (Fig. 3C–E, Fig. S3). Because the GUV system is in thermodynamic equilibrium and lacks the actin cytoskeleton and lipid domains, the observed redistribution supports the idea that proteins spontaneously re-organise at cell–cell contacts largely according to size (Carbone et al., 2017; Chang et al., 2016; Schmid et al., 2016), irrespective of the size of the contact (down to <250 nm).

These considerations suggest that strong apposing forces from antigen-presenting surfaces may not be required for receptor reorganisation at close contacts, as GUVs and LUVs are soft and exhibit little resistance to deformation (Dimova, 2014; Fenz and Sengupta, 2012). To further confirm that the observed reorganisation was independent of signalling, we expressed a non-signalling form of rat CD48 (rCD48) on Jurkat cells and presented rat CD2 (rCD2) on the GUV surface. This interaction was used to form a close contact de-coupled from signalling in the Jurkat cells. We observed clear CD45 exclusion at contacts showing that signalling is not required to initiate CD45 exclusion (Fig. S3).

### Lymphocyte signalling induced by cell–GUV contact

**Fig. 4.**
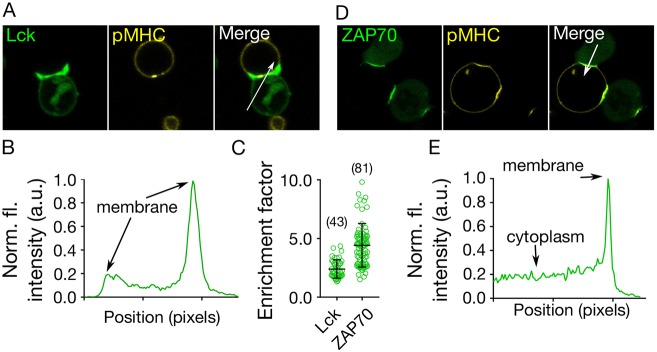
**GUV-induced activation of T cells.** (A) Cellular localisation of Lck (labelled with EGFP, green) following T cell binding to GUVs coated with pMHC. (B) The intensity line profile of the Lck fluorescence signal through the contact (arrow in A) shows enrichment of Lck at the contact. (C) Enrichment of Lck/ZAP70 at GUV–cell contact sites. The enrichment factor represents the ratio of fluorescence intensity at the contact site versus non-contact site (cytoplasmic signal for ZAP70). (D) Cellular localisation of ZAP70 (labelled with HaloTag™) upon binding to vesicles carrying pMHC. (E) The intensity line profile of the ZAP70–HaloTag fluorescence signal through the contact zone (arrow in D) shows enrichment at the contact (image size 40 µm×40 µm). Error bars represent standard deviation of the mean. Data is representative of at least three independent experiments and the number of data points is indicated on the graphs in parentheses.

During lymphocyte activation, downstream-signalling tyrosine kinases are recruited to triggered receptors. In T cells, the principal kinase is ZAP70, and in mast and B cells it is Syk (Brownlie and Zamoyska, 2013; Wernersson and Pejler, 2014). To test whether signalling could be induced in the GUV–cell contact, we generated T cell lines expressing EGFP- and mNeonGreen-tagged forms of Lck and ZAP70, respectively. After incubating cells expressing Lck–EGFP with GUVs presenting pMHC, we observed enrichment of the Lck kinase in the region of contact (Fig. 4A–C, Fig. S4). At the GUV–cell contact we also observed the recruitment of ZAP70 (Fig. 4C–E, Fig. S4). Cognate pMHC on the GUV surface was required to recruit ZAP70 to the membrane, as indicated by the observation that pMHC comprised of a peptide not recognised by the 1G4 TCR (derived from the melanoma antigen gp100) produced no increase in mNeonGreen–ZAP70 fluorescence (Fig. S4). Moreover, when CD58 alone was presented on the GUV surface, ZAP70 was not recruited to the membrane following stable contact formation (Fig. S4). ZAP70 recruitment is a robust indicator of TCR triggering, and our data therefore show that the free-standing *in vitro* system is capable of activating T cells.

**Fig. 5.**
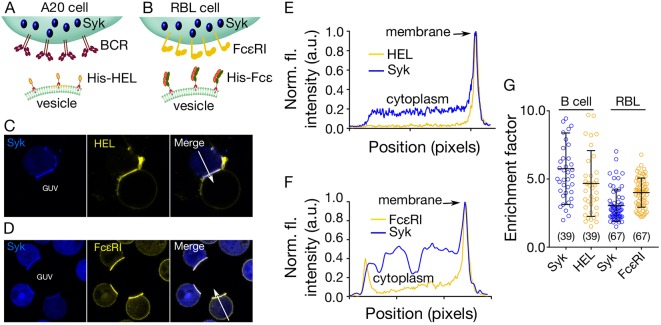
**Mast and B cell activation initiated by GUV**–**cell contact.** (A) Diagram showing the *in vitro* system used for studying A20 (B cell) signalling. Syk kinase is tagged with mCitrine in A20 cells and HEL labelled via a HALO^®^ tag and presented on the GUV surface. (B) Diagram showing the *in vitro* system used for studying RBL-2H3 (mast cell) signalling. Mast cell Syk kinase was mCitrine-tagged, and FcεRI was fluorescently labelled via a SNAP^®^ tag. GUVs presented a His-tagged form of the Fcε portion of the IgE antibody, which served as a ligand for FcεRI. (C,D) Example images of cellular localisation of Syk and either HEL (C) or FcεRI (D) in A20 or RBL-2H3 cells upon contact with GUVs (image size 40 µm×40 µm). (E) Intensity line profile of the Syk and HEL fluorescence signal through the contact (white arrow in C). (F) Intensity line profile of the Syk and FcεRI fluorescence signal through the contact (white arrow in D). (G) Quantitation of HEL/FcεRI and Syk kinase at GUV–cell contact sites. The enrichment factor represents the ratio of fluorescence intensity at the contact versus non-contact sites. Error bars represent standard deviation of the mean. Number of data points obtained from at least three independent experiments are indicated on the graphs in parentheses.

To investigate signalling in a system not based on T cells, we also tested whether we could visualize BCR signalling in B cells, and FcεRI triggering in mast cells, induced by GUVs presenting receptor ligand mimics. For this, we used a mouse B cell line (A20) and a rat basophilic leukaemic cell (RBL-2H3; i.e. mast cells), expressing Syk kinase tagged with mCitrine fluorescent protein. To create model cell contacts, the GUVs were reconstituted with either a His-tagged form of hen egg lysozyme (His–HEL), which binds to the HyHEL10 BCR (Fig. 5A), or the His-tagged Fc region of rat IgE antibody (His–Fcε), which binds FcεRI expressed by mast cells (Fig. 5B). His–HEL and FcεRI were labelled via HALO and SNAP tags, respectively. When incubated with the GUVs, both the A20 and RBL-2H3 cells showed similar robust recruitment of the Syk kinase to contacts where the receptor was also enriched (Fig. 5C–G, Fig. S5). Unspecific His-tagged Fab (human CD45 Fab) on the GUVs did not trigger Syk recruitment or receptor enrichment (Fig. S5).

## DISCUSSION

*In vitro* reconstitution-based technologies are finding widespread use for analysing the biophysical basis of complex cellular processes (Jørgensen et al., 2017; Liu and Fletcher, 2009). GUVs have found applications in a broad range of fields, probing many aspects of cell biology (Bhatia et al., 2018; Dubavik et al., 2012; Kahya, 2010; Prevost et al., 2017; Richmond et al., 2011; Sezgin et al., 2015a). The first obvious advantage of GUV-based systems for disentangling the complexity of cell–cell interactions is that the 3D topology of a contact can be studied. This is especially important given the prominent role being assigned to microvilli-based contacts among immune cells (Jung et al., 2016). A second advantage is that the complexity and membrane properties of GUVs are highly tuneable through the modular insertion of membrane proteins and by varying their lipid composition. The use of more complex lipid mixtures or reconstitution of the actin cytoskeleton could provide the means to modify the stiffness of GUVs. Substrate stiffness is increasingly being studied as a factor in understanding immune cell activation (Beningo and Wang, 2002; Saitakis et al., 2017), therefore the GUV-based approach could provide important new insights into receptor signalling. A third advantage is the finite size of GUVs, which makes them better mimics of cells than SLBs. Fourth, vesicular systems allow, in principle, the reconstitution of full-length membrane proteins which, unlike SLBs, opens up the possibility of studying the immediate sequelae of receptor triggering.

Here, we present a free-standing GUV-based membrane system capable of yielding important insights into the spatiotemporal basis of immune cell–cell interactions and lymphocyte activation. We first showed that the GUV format allows the essentially unhindered diffusion of small and large proteins in the bilayer, giving diffusion constants substantially larger than those we obtained for SLBs. We confirmed that it was possible to “fine-tune” protein mobility by varying the lipid composition of the GUVs. We also showed, by inserting key cell-surface proteins expressed by antigen-presenting cells into GUVs, and by imaging the contacts formed between the GUVs and live T cells, that we could readily observe the patterns of large-scale spatial reorganisation of key surface proteins previously seen using supported surfaces (Cai et al., 2018; Chang et al., 2016; Dustin and Choudhuri, 2016; Dustin and Groves, 2012; Dustin et al., 2007; Varma et al., 2006). We observed the co-enrichment of small adhesion proteins (e.g. CD58) and activating ligands (e.g. pMHC) and the exclusion of large molecules (ICAM-1) and inhibitory proteins (CD45) at GUV–T cell contacts. Although we observed stronger exclusion of CD45 compared with ICAM-1, we did not observe the formation of separate dSMACs and pSMACs (i.e. distal and peripheral regions of the contact in which CD45 and ICAM-1 accumulate, respectively), consistent with dSMAC and pSMAC being formed via active rather than passive processes (Freiberg et al., 2002; Johnson et al., 2000). Our minimal system was sufficient to induce at least the early stages of T cell activation, as seen by the recruitment of downstream signalling effectors to the contact. We made highly analogous findings for B cells and mast cells triggered with BCR and FcεR ligands presented by GUVs, respectively, reinforcing the apparent similarities between leukocytes, at least with respect to the earliest stages of signalling.

We confirmed that the reorganisation of surface proteins occurred independently of cytoskeletal effects or the influence of lipid organisation in the GUVs, supporting previous reports that receptor–ligand binding energy and the size-dependent lateral segregation of proteins are responsible for the observed patterning of molecules (Carbone et al., 2017; James and Vale, 2012; Schmid et al., 2016). Importantly, we observed contact formation, and molecular re-organisation and signalling, all in the ‘softest’ GUVs we could produce (Dimova, 2014), implying that very low levels of force, if any, are needed to affect the early signalling at cell–cell contacts, contrary to other reports (Brazin et al., 2015; Das et al., 2016). Although the reorganisation of CD58 and pMHC could, in principle, be dependent on active processes occurring on the T cell side of the contact, the behaviour of CD45 attached to GUVs was not, as there are no known ligands for CD45.

Recent studies indicate that membrane topology may have an important role in the very earliest stages of T cell activation (Cai et al., 2017; Razvag et al., 2018). T cell microvilli, which are ‘finger-like’ projections of ∼1 µm length and ∼100–500 nm diameter (Cai et al., 2017; Jung et al., 2016), are used to scan APCs for activating pMHC, providing a small, spatially defined region within which antigen discrimination must take place. Mimicking these spatially limited T cell microvilli–APC contact sites should be useful in attempts to understand the signalling consequences of constraining T cell–APC contact geometrically in this way. For example, there are differing reports as to whether CD45 is present (Cai et al., 2017), depleted (Jung et al., 2016) or locally segregated (Razvag et al., 2018) from microvilli tips. As proof-of-principle, we show here that LUVs, which can be fractionated to produce homogenous vesicles in the range of 100 nm to 1 µm diameter (Lapinski et al., 2007), allow the generation of model cell contacts in the size range likely generated by microvilli. We observed robust spontaneous exclusion of the phosphatase at contacts formed with LUVs of <250 nm diameter. One explanation for this could be that CD45 is excluded less efficiently at real cell–cell contacts than for the compositionally less-complex LUVs we are presently studying. We note that the size-tunability of unilamellar vesicles could allow mimics of smaller structures to be created, such as microorganisms, organelles, pollen grains and lipid-enclosed viruses.

It is important, finally, to acknowledge the disadvantages of the GUV-based model system for studying cell–cell contacts. Because the vesicles are free standing, they are not immobile over long-enough periods to allow hour-long measurements needed, for example, for super-resolution imaging. Also, certain imaging technologies cannot be applied to GUVs, such as atomic force microscopy (AFM). Despite these disadvantages, which surely are not insurmountable, free-standing GUVs offer a powerful tool for dissecting cell surface biology both within and outside the immune system.

## MATERIALS AND METHODS

### Lipids and proteins

POPC, Brain PC, DPPC, DGS-Ni-NTA and cholesterol were obtained from Avanti Polar Lipids. cDNA encoding ECD fragments of CD45 (CD45RABC, residues 24–575, UniProtKB P08575), CD58 (residues 29–215, UniProtKB P19265), CD54 (ICAM-1, residues 28–480, UniProtKB P05362) and CD2 (ratCD2, residues 23–219, UniProtKB P08921) were ligated into pHR downstream of the sequence encoding cRPTPσSP, having a H_6_SRAWRHPQFGGH_6_ ‘spacer-His’ tag on the C-terminus. Soluble protein expressed by lentiviral transduction in 293T cells was purified using metal-chelate and size-exclusion chromatography. Soluble pMHC (HLA-A) was produced as previously described (Altman et al., 1996). For the purpose of labelling CD45, a Fab digested from the whole antibody clone Gap8.3 tagged with Alexa Fluor 488 was used.

### Preparation of SLBs, GUVs and LUVs

SLBs were prepared using a spin-coating method (Clausen et al., 2015). Glass coverslips (#1.5) were first cleaned with piranha solution (sulfuric acid and hydrogen peroxide, 3:1) for 45 min. After washing the coverslips with distilled water, 1 mg/ml lipid mixture (POPC:DGS-Ni-NTA, 96:4 molar ratio) was spread on them. Immediately after, they were spun at 3000 rpm for 40 s. Dried lipid bilayer was hydrated with SLB buffer (150 mM NaCl, 10 mM HEPES, pH 7.4). After formation, SLBs were incubated with 1 µg/ml His-tagged protein for 30 min. Then, they were washed 10 times by adding and removing fresh buffer.

GUVs were prepared using an electroformation method. Lipid mixture (1 mg/ml POPC:DGS-Ni-NTA, 96:4 molar ratio) was deposited on platinum wire and dried. It was then dipped into a Teflon-coated chamber filled with 300 mM sucrose. A 10 Hz AC field for 1 h followed by 2 Hz for 30 min triggered GUV formation. After formation, 100 µl of the GUV suspension was incubated with 1 µg/ml His-tagged protein for 30 min. To wash out unbound protein, the GUV mixture was gently mixed with 1 ml PBS and allowed to sediment for 30 min. The bottom 100 µl was transferred to a new tube containing 1 ml PBS. This process was repeated twice. GUVs were imaged in PBS as described in a later section.

LUVs were prepared as previously reported (Sezgin et al., 2014). Briefly, lipid mixture was first dried under N_2_ gas. Later, it was re-suspended in buffer containing 150 mM NaCl and 10 mM HEPES. After 1 min vortexing, the suspension was sonicated for 30 min in a water bath sonicator. His-tagged proteins were added to the suspension at 1 µg/ml concentration. After 30 min of incubation with proteins, liposomes could be used directly for the experiment. Although the small amount of unbound proteins does not affect liposome binding to the cells, if wanted, liposomes can be washed by centrifugation. LUVs can be pelleted by centrifugation at 6700 ***g*** for 30 min. During this process, there is a significant loss of smaller liposomes that are generated during centrifugation because smaller vesicles (small unilamellar vesicles; SUVs; <100 nm) cannot be pelleted, even at >100,000 ***g***. However, centrifugation of LUVs at higher ***g***-force might deform them.

### Cell lines

Jurkat-derived T cell lines (ATCC TIB-152) were cultured in sterile RPMI supplemented with 10% FCS, 2 mM l-glutamine, 1 mM sodium pyruvate, 10 mM HEPES and 1% penicillin-streptomycin-neomycin solution. Zap70 was labelled with Halo tag and Lck labelled with eGFP. RBL-2H3 cells (ATCC CRL-2256) were cultured in minimum essential medium Eagle (MEM; Sigma-Aldrich) supplemented with 10% FCS, 1 mM L-glutamine (Sigma-Aldrich). At 24 h before imaging, cells were incubated in Falcon tubes overnight on an end-over-end rotator (6 rpm) at 37°C. FCεRI was labelled with SNAP tag and Syk labelled with mCitrine. A20-derived B cell lines (ATCC TIB-208) were cultured in sterile RPMI supplemented with 10% FCS, 2 mM L-glutamine, 1 mM sodium pyruvate, 10 mM HEPES, 1% penicillin-streptomycin-neomycin solution and 0.5 mM 2-mercaptoethanol. HyHEL10 was labelled with SNAP tag and Syk labelled with mCitrine. All cells were maintained at 37°C and 5% CO_2_ during culturing.

Halo and SNAP labelling was carried out using Oregon green-Halo (NEB), SNAP-Cell^®^ TMR-Star (NEB), HaloTMR (Promega) or JF-646 (gift from Janelia Farm laboratories). Cells were incubated with 0.1 mM (final concentration) of the dyes at 37°C for 30 min. Afterwards, they were spun down at 1500 rpm for 3 min. Then, they were washed by re-suspending in pre-warmed fresh medium (with all the supplements) and spinning down again. Later, fresh medium was added and they were incubated for another 30 min to remove unbound dyes inside the cells.

For cellular CD45 labelling, ∼10^6^ cells were incubated with anti-CD45 (Gap8.3) Alexa Fluor 488-labelled Fabs (degree of labelling ∼2 moles of dye per mole of protein) diluted in 100 µl of HEPES buffer at a final concentration of 10 nM at 37°C for 15 min.

### Fluorescence correlation spectroscopy

FCS on the GUVs and SLBs was carried out using a Zeiss 880 microscope, 40× water immersion objective (numerical aperture 1.2) as described before (Schneider et al., 2017). Briefly, before measurement, the shape and size of the focal spot were calibrated using Alexa Fluor 488 and 647 dyes in water in an eight-well glass bottom (#1.5) chamber. To measure diffusion on the membrane, the SLBs formed on #1.5 glass or GUVs placed into an eight-well glass bottom (#1.5) chamber were used. The laser spot was focused on the membrane by maximising the fluorescence intensity. Then, three curves were obtained for each spot (5 seconds each). The obtained curves were fit using the freely available FoCuS software (Waithe et al., 2015).

### Confocal and spectral generalised polarisation imaging

After GUVs were gently transferred to an eight-well Ibidi chamber filled with 250 µl PBS, 50 µl of cells suspended in Fluorobright medium (a low fluorescence version of standard DMEM medium) was added into the wells. The imaging was performed at 37°C in PBS. The samples were imaged with a Zeiss LSM 780 or 880 confocal microscope. Pacific Blue was excited with 405 nm and emission collected with band pass 420–480 nm. Alexa Fluor 488-labelled proteins were excited with 488 nm and emission collected at 505–550 nm. Alexa Fluor 555-labelled proteins were excited with 543 nm and emission collected at 570–630 nm. Molecules labelled with Alexa Fluor 647 were excited with 633 nm and emission collected with a LP 650 filter. Multitrack mode was used to eliminate the cross-talk between channels. Images were later analysed in Fiji/ImageJ (Schindelin et al., 2012). For 3D images, Imaris (Oxford Instruments) was also used.

Spectral imaging was performed on a Zeiss LSM 780 confocal microscope equipped with a 32-channel GaAsP detector array. Laser light at 405 nm was selected for fluorescence excitation of Laurdan. The lambda detection range was set between 415 and 691 nm for Laurdan. The images were saved in .lsm file format and then analysed using a freely available plug-in compatible with Fiji/ImageJ, as described previously (Sezgin et al., 2015b), which can be found at http://github.com/dwaithe/GP-plugin.

## Supplementary Material

Supplementary information
