# Supplementary material for: Reconstitution of immune cell interactions in free-standing membranes
Source: J Cell Sci. 2018 Oct 2;132(4):jcs219709. doi: 10.1242/jcs.219709 (PMC6398472; doi:10.1242/jcs.219709)
Supplement: Supplementary information [file joces-132-219709-s1.pdf]

Supplementary Figures

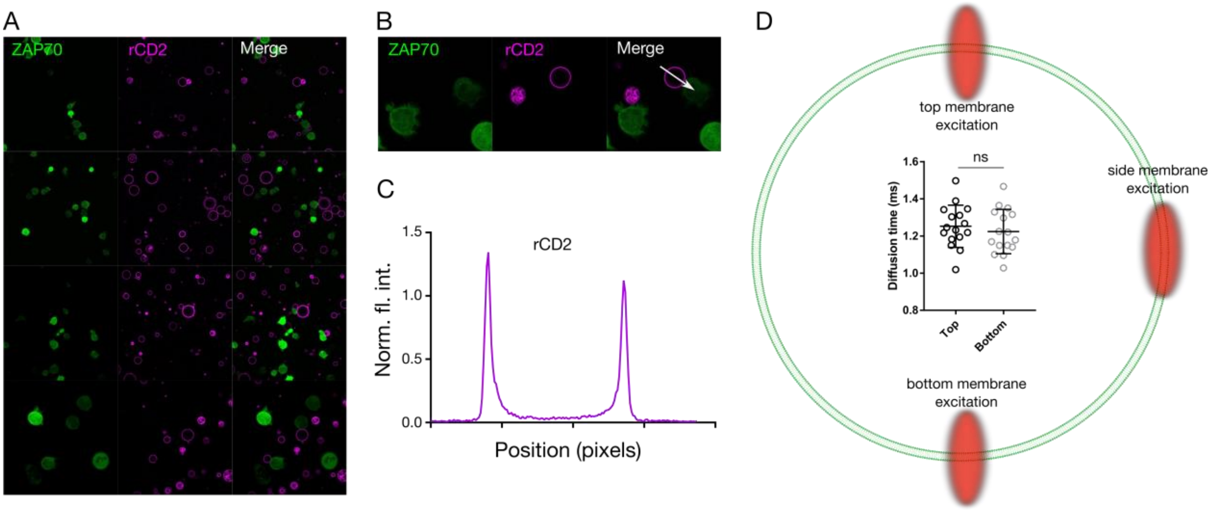

Figure S1. No non-specific recruitment of receptors between GUV and cells. GUVs decorated with Alexa-647 labelled rat CD2 (rCD2) were incubated with Zap70-eGFP^{+} Jurkats. As these cells do not express rat CD48 (rCD48), specific contacts with GUVs will not occur. Rarely appearing unspecific pseudo-contacts seen between rCD2-GUVs and Jurkats are not bona fide real contacts. They do not accumulate rCD2 (shown in **B** & **C**). (**A & B**) Confocal image of Zap70-eGFP+ Jurkats (left), Alexa-647 labelled rCD2-GUVs (middle) and these merged (right). (**B**) Rare pseudo-contact formed between GUV and Jurkats. (**C**) Line profile of labelled rCD2 for the white line shown in **B**. Left peak and right peak indicate non-contact and 'pseudo-contact' zone with Jurkat, respectively showing no accumulation of rCD2 or Zap70 on the contact side. **(D)** FCS measurements on GUVs. Confocal spot was placed either at the top or the bottom surface of the GUVs. The diffusion time of His-tagged GFP does not show any difference between top or bottom measurements (inset graph). Measurements at the side membrane is not accurate since the portion of the membrane is larger which can alter the time molecules spend in the focal volume.

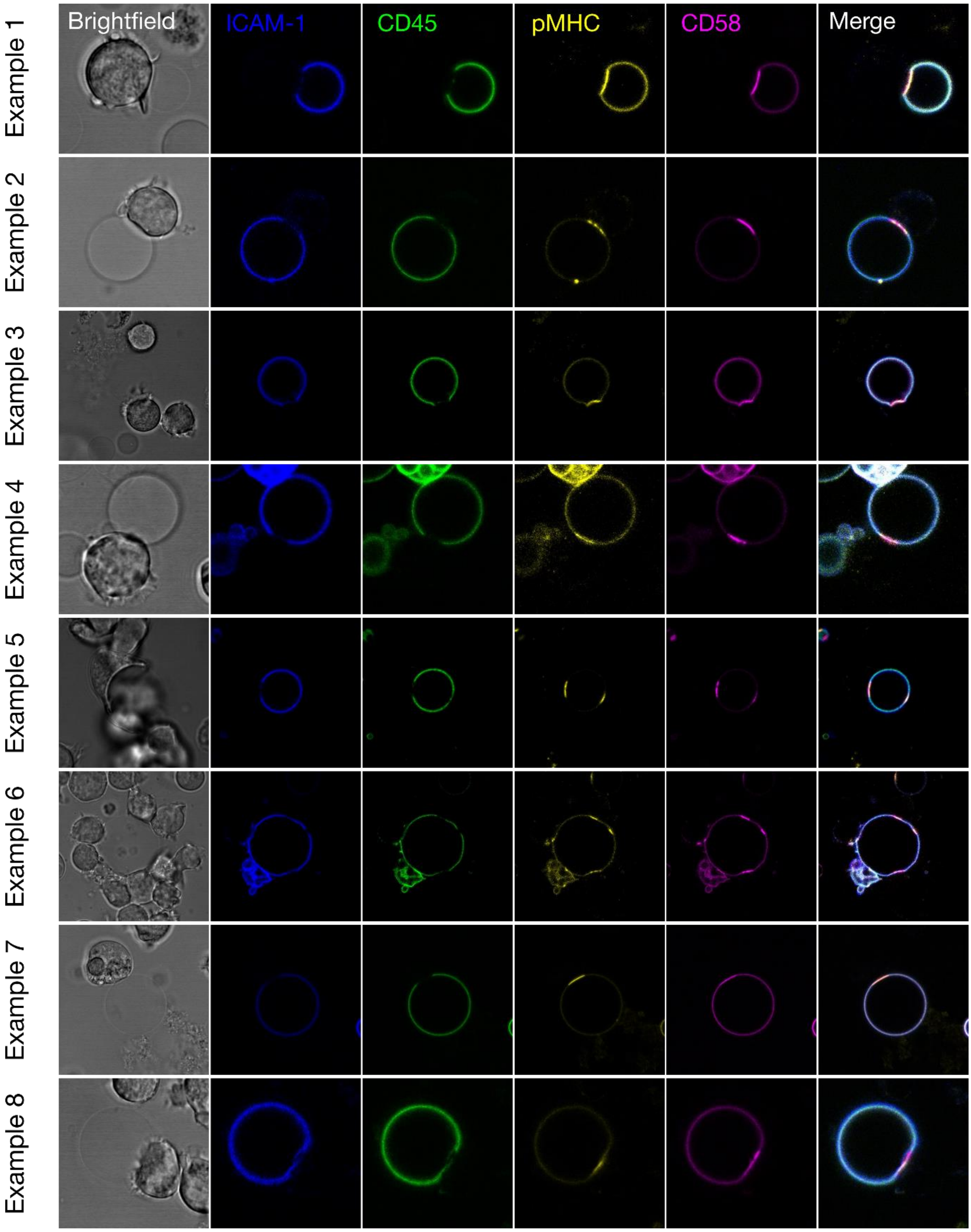

Figure S2. More examples of receptor reorganisation at GUV-cell contacts. Nickelated GUVs loaded with His-tagged ICAM-1, CD45, pMHC (9V) and CD58 were incubated with 1G4^{+}-Jurkat cells for 5 minutes to allow sufficient time to form contacts (image sizes are 40 µm x 40 µm). Larger proteins (ICAM and CD45) segregate away from the GUV-cell contacts formed from small proteins (pMHC and CD58). Image sizes are 40 µm x 40 µm.

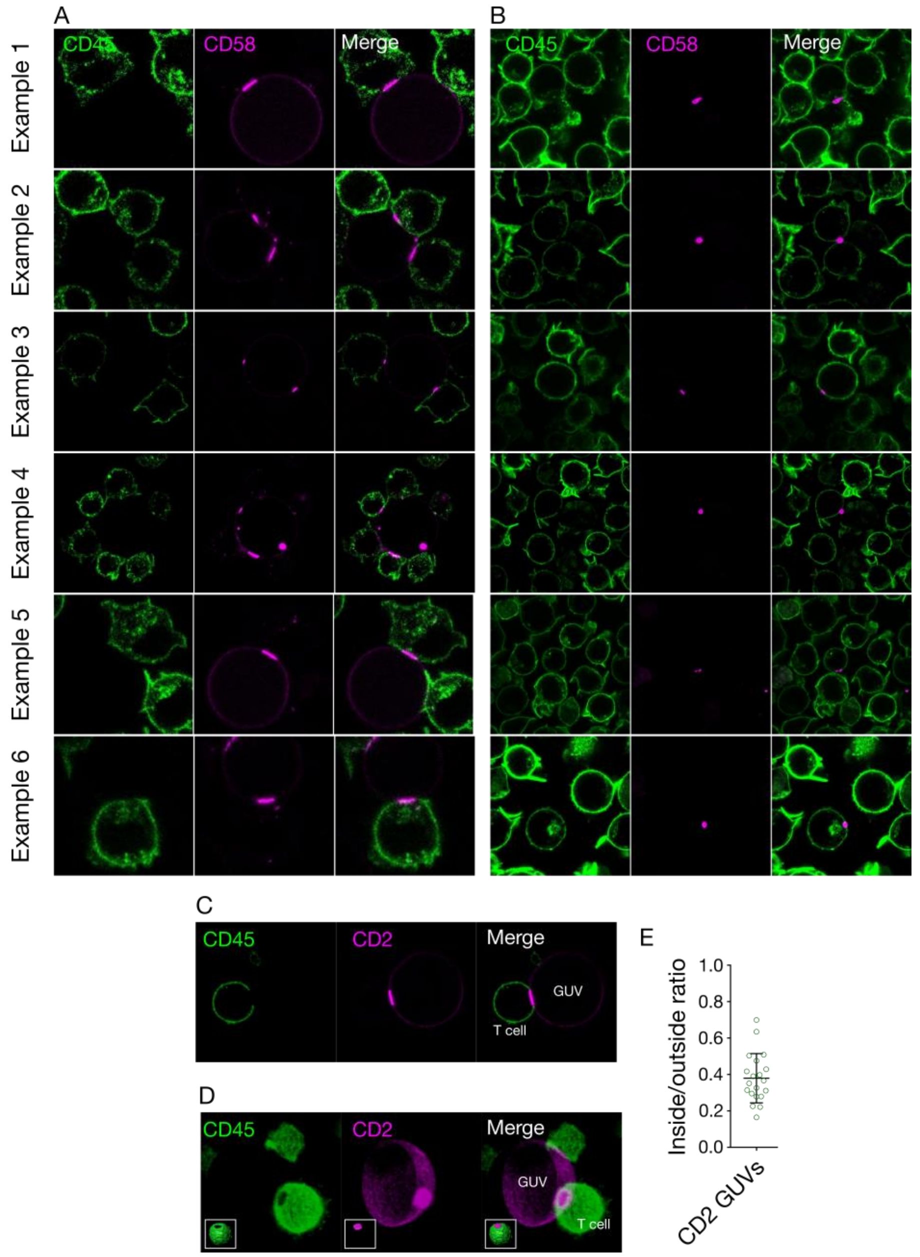

Figure S3. Requirements for CD45 segregation. (A, B) More examples of CD45 segregation using CD58 containing GUVs and liposomes. (**A**) CD58-GUVs, or (**B**) CD58-liposomes, both purple, in contact with CD45 labelled Jurkats (green). CD45 was labelled using anti-CD45 Gap8.3 Fab-Alexa488. CD45 intensity is diminished at GUV-cell contact sites in both **A** and **B.** Image sizes are 40 µm x 40 µm for A and 50 µm x 50 µm for B. **(C-E)** Signalling-deficient adhesion induces segregation of CD45. Signalling deficient variant of rCD48 was stably introduced into Jurkats. Cells were labelled using anti-CD45 Gap8.3 Fab-Alexa488 and incubated with rCD2-GUVs. (**C**) 2-D and (**D**) 3-D plot of cell-GUV interaction. (**E**) Ratio plot of CD45 intensity inside/outside GUV-cell contact (n=20). Image sizes are 40 µm x 40 µm. Error bars represent standard deviation of the mean.

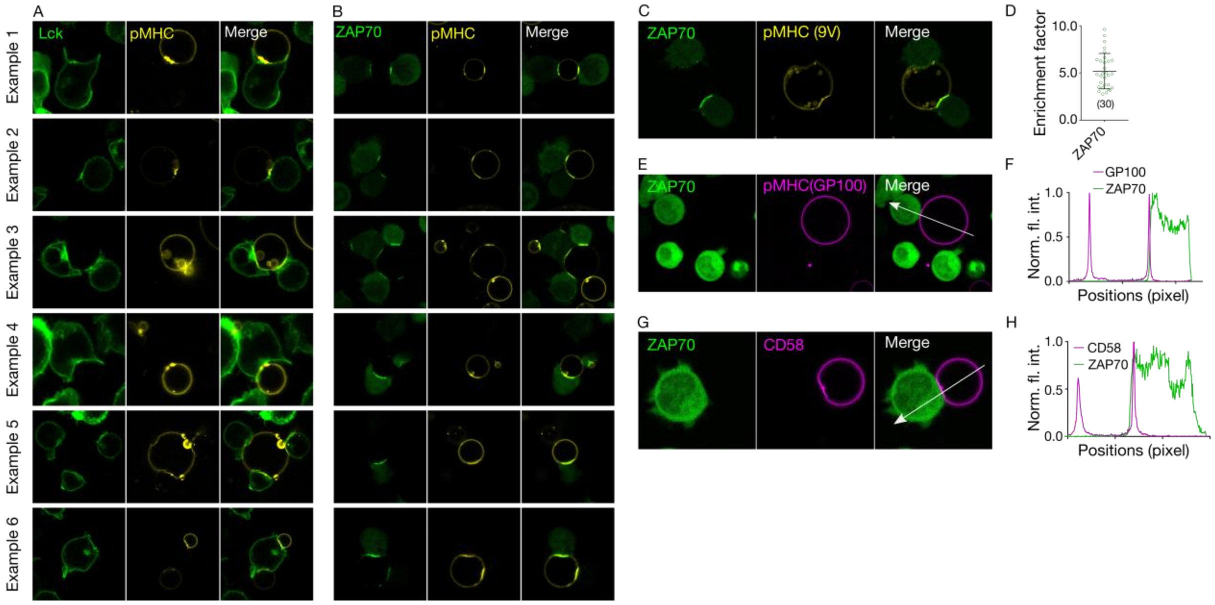

Figure S4. Lck and ZAP70 recruitment to GUV-cell contact site. Several examples of (**A**) Lck or (**B**) ZAP70 recruitment to the GUV-cell contact site. This recruitment only coincides at sites of pMHC enrichment indicating signalling driven recruitment. Image sizes are 40 µm x 40 µm. **(C-H)** ZAP0 recruitment with MHCI (9V), MHCI (GP100) and CD58 alone. (**C**) ZAP70 is recruited to the contact zone when only MHC1 is present. (**D**) Quantification of ZAP70 recruitment to the contact zone. (**E**) There is no notable ZAP70 signal at the membrane when pMHCI carries a peptide that TCR cannot recognise (GP100) on the GUV surface. **(F)** Intensity profile of the line shown in panel E, showing no enrichment of pMHCI(GP100) or ZAP70 in the contact area. (**G**) There is no notable ZAP70 signal at the membrane when only CD58 is present on the GUV surface. **(H)** Intensity profile of the line shown in panel G, confirming enrichment of CD58 in the contact area but not of ZAP70. Image sizes are 40 µm x 40 µm. Error bars represent standard deviation of the mean. Data is representative of at least 3 independent experiments (N=30). Image sizes are 40 µm x 40 µm.

Journal of Cell Science • Supplementary information

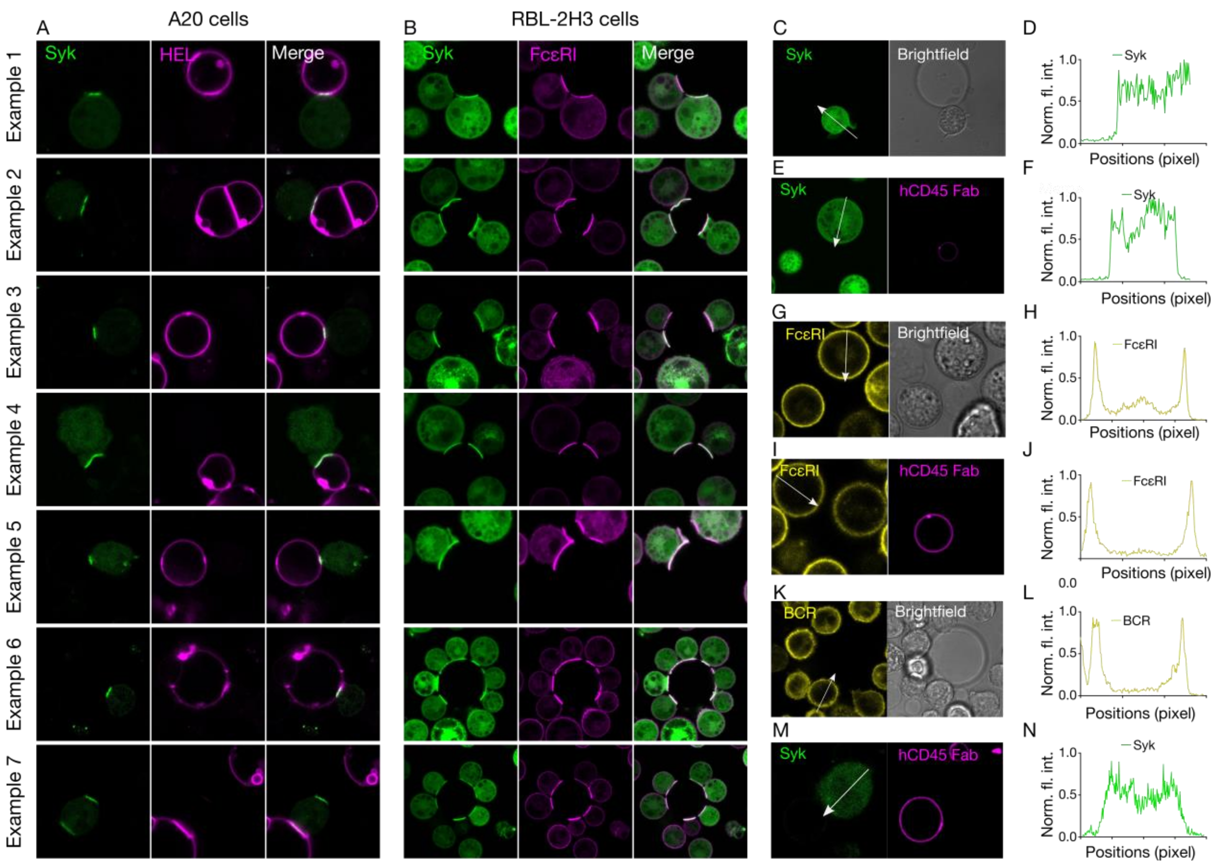

Figure S5. Control experiments for RBL-2H3 and B cells. (A, B) Several examples of (**A**) A20 (B-cells) or (**B**) RBL-2H3 cells forming contact with GUVs. The contact zone recruits receptor and the kinases in the contact area. Image sizes are 40 µm x 40 µm (60 µm x 60 µm for example 6 and 7 RBL-2H3 cells). **(C-J)** Activation of RBL-2H3 cells in the absence of specific protein: (**C**) There is no notable Syk signal at the membrane when no protein is presented on GUV surface. (**D**) Intensity profile of the line shown in panel C, confirming no recruitment of Syk. (**E**) There is no notable Syk signal at the membrane when unspecific Fab (anti-human CD45 Fab) protein is presented on GUV surface. (**F**) Intensity profile of the line shown in panel E, confirming no recruitment of Syk. (**G**) There is no notable FcεRI enrichment at the contact zone when no protein is presented on GUV surface. (**H**) Intensity profile of the line shown in panel G, confirming no FcεRI enrichment. (**I**) There is no notable FcεRI enrichment at the contact zone when unspecific Fab (anti-human CD45 Fab) protein is presented on GUV surface. (**J**) Intensity profile of the line shown in panel I, confirming no FcεRI enrichment. Image sizes are 40 µm x 40 µm. **(K-N)** Activation of A20 cells in the absence of specific protein. (**K**) There is no notable BCR enrichment at the contact zone when unspecific Fab (anti-human CD45 Fab) protein is presented on GUV surface. (**L**) Intensity profile of the line shown in panel K, confirming no FcεRI enrichment. (**M**) There is no notable Syk signal at the membrane when no protein is presented on GUV surface. (**N**) Intensity profile of the line shown in panel N, confirming no recruitment of Syk. Image sizes are 40 µm x 40 µm.
